# Mating marks on museum specimens reveal breeding patterns in species of *Pterostichus* Bonelli (Carabidae, Pterostichini)

**DOI:** 10.3897/BDJ.9.e70897

**Published:** 2021-09-24

**Authors:** Kipling Will, Patina K. Mendez

**Affiliations:** 1 University of California, Berkeley, ESPM Dept., Berkeley, CA, United States of America University of California, Berkeley, ESPM Dept. Berkeley, CA United States of America; 2 Essig Museum of Entomology, University of California, Berkeley, Berkeley, CA, United States of America Essig Museum of Entomology, University of California, Berkeley Berkeley, CA United States of America

**Keywords:** Mediterranean-type climate, life history, mating

## Abstract

We found distinct and consistently placed, species- and sex-specific abrasions of the cuticle on museum specimens of 14 species of the *Pterostichus* Bonelli, 1810 (Carabidae, Pterostichini) subgenusHypherpes Chaudoir, 1838. We deduced that these marks are generated during mating and, therefore, can be used to distinguish between preserved specimens of beetles that had previously mated at the time of capture and those that had not mated. In addition to describing and detailing the occurrence of the marks and providing evidence that they are the result of mating, we demonstrate their utility for inferring life history using a museum voucher collection. By scoring these indications of mating from pinned specimens, we describe life cycle patterns in two similar, relatively closely related and sympatric species of the subgenus *Hypherpes, P.vicinus* Mannerheim, 1843 and *P.californicus* (Dejean, 1828). Both were sampled during a pitfall trap study in Contra Costa, California, USA from 2014–2019 and deposited in the Essig Museum of Entomology, UC Berkeley. Both species had very low adult activity through the drought and end of drought period prior to the spring of 2017 and are significantly more abundant in the post-drought period. Based on mating marks, both species responded to accumulated precipitation ending the drought by the emergence of an active, mostly unmated cohort of adults. The spring activity peak, following the end of the drought, was dominated by unmarked and presumably unmated beetles, but samples from subsequent springs included a nearly equal mix of beetles showing mating marks and apparently unmated beetles. The beetle activity appears to correspond more with the accumulated rainfall of the preceding rainy season than with the rains of the sample year. Beetles sampled in autumn and winter (rainy season) predominantly show mating marks. The occurrence throughout the year of beetles that are marked as having mated is consistent with iteroparous beetles with a lifespan of more than one year and also consistent with dynamic phenotypic polyvariance in which the adult activity period is synchronised by adjusting development time. The dominant pattern fits with a life cycle that is typically annual univoltine, or possibly biennial semivoltine in dry years, rainy season breeding (autumn-winter) iteroparous, with adult summer aestivation and possibly facultative larval hibernation. However, unmarked and so apparently unmated individuals and teneral adults were captured during peak activity periods regardless of the season, suggesting that either the beetles diapause as teneral adults that then complete development and become active at various points during the year and/or there are multiple periods of breeding and oviposition each year in at least some portion of the population.

## Introduction

There is a wealth of publications on life-history traits of carabids, albeit based almost entirely on species from middle-and high-latitude temperate regions that experience cool to cold winters and significant precipitation throughout most of the year (e.g. Kaufmann 1971, Bousquet 1986, Castro et al. 2014, Lindroth 1985, Lindroth 1986, see also various summary publications, den Boer and van Dijk 1998, Thiele 1977, Matalin 2007, Nolte et al. 2019, Kotze et al. 2011, Homburg et al. 2013) with a few exceptions (e.g. Paarmann 1970, Paarmann 1973, Paarmann 1974). For North American pterostichines, life cycle observations are mostly from works by Bousquet (Bousquet 1986, Bousquet 1999) who detailed 12 north-eastern species and provided a summary of what is known of the life history of the group in North America. Other publications on North American pterostichines also propose breeding patterns based on adult activity, the occurrence of teneral individuals or observations of apparent mating in the field (e.g. Bergdahl and Kavanaugh 2011, Bousquet 1999). Typically these publications use the terms “autumn breeder” and “spring breeder,” a system introduced by Larsson (1939) to describe carabid life cycles. In this system, spring breeders have an adult activity peak in spring when mating occurs, oviposition in late spring and summer, larval development in summer, a second adult activity peak in autumn when feeding, but not mating occurs and adults hibernate in winter. Autumn breeders have a single adult activity peak in summer into autumn when mating occurs, oviposition happens in late summer to early autumn, larval development and hibernation occur through the following spring.

Bousquet (1999) and others (e.g. Lindroth 1949, den Boer and van Dijk 1998, Matalin 2007, Makarov 1994, den Boer and den Boer-Daanje 1990) correctly pointed out that this seasonal-breeder designation is a simplification that does not capture life cycle variation across carabids well. Attempts have been made to explore alternative life cycle descriptions. For example, Lindroth (1949) focused on the hibernation period of the larvae; however, this proved unsatisfactory since most carabids appear to be univoltine and iteroparous (den Boer and van Dijk 1998, Matalin 2007) with larvae and adults frequently overwintering, typically in hibernation. Given the challenge of categorising breeding cycles in the relatively predictable seasons of the wet, north temperate, it is no surprise that California’s Mediterranean-type climate, with its relatively warm to hot temperatures all year, seasonal rains with high inter-annual variability and frequent multi-year droughts punctuated by wildfires (Seager et al. 2019), would provide a venue for life cycles that are not well-described by the canonical spring breeder/autumn breeder classification. In addition, the Mediterranean-type climate’s high inter-annual variability requires an extended sampling period that includes wetter and drier years to build a full picture of insect life cycles and their potential plasticity.

Previous studies of the bionomics of pterostichine carabid beetles relied on extensive dissections to study soft tissues, such as ovarioles and testes, not typically preserved in pinned specimens (Bousquet 1986). Seasonal changes in abundance of adults and the appearance of teneral adults can also be scored from pinned collections to provide a slightly coarser picture of the life history of a given species. We found, somewhat serendipitously, that museum specimens can provide more information on the breeding status than previously known.

Natural history museum collections have always been a critical part of biological research as they provide the inspiration behind questions and the data to answer them (Bakker et al. 2020, Holmes et al. 2016, Suarez and Tsutsui 2004). Voucher specimens from a given study are not just static objects in collections, but are also frequently re-examined for a purpose entirely different from the original study for which they were collected. While examining carabid beetle voucher material, we found an abrasion on the cuticle that indicates that individuals had mated. As far as we know, an external indication of mating status for both sexes, based on non-genitalic morphology, has not been previously documented in any insect group. However, we anticipate that careful inspection of museum specimens will reveal similar alterations related to insect mating in other carabids and other insects. The marks left externally on female hemipterans and strepsipterans and internally in some drosophilid flies as a result of traumatic insemination by males (Tatarnic et al. 2014) are analogous and potentially would allow for the study of breeding patterns of females using museum specimens.

We document these mating marks in some pterostichine carabid beetles and to demonstrate their utility, use them to investigate the life histories of two mark-bearing species, *Pterostichusvicinus* Mannerheim, 1843 and *P.californicus* (Dejean, 1828), represented by museum voucher specimens from a long-term pitfall trap study conducted in the Diablo Range of California (Will and Mendez, unpubl. data). Furthermore, we compare their phenology and the phenologies of two other, sympatric *Pterostichus* species collected during the study, P. (Hypherpes) protensiformis (Casey, 1924) and P. (Leptoferonia) angustus (Dejean, 1828). The catch numbers, the occurrence of tenerals and the presence of mating marks are compared to the rainfall record in order to determine potential seasonal activity-inducing tokens in the environment and examine the beetles’ response to the end of a multi-year drought.

## Material and methods

### Field site and sampling methods

The arthropod vouchers, from which the pterostichines for this study are drawn, were collected during a long-term ecological study of the impact of wildfire and drought on epigean arthropods (Will and Mendez, unpubl. data) in Perkins Canyon (vicinity of N37.89587, W121.87486), Mt. Diablo State Park, Contra Costa County, California, USA. Beetles were collected in a 60­–trap array of pitfall traps using standard 16 oz. (473 ml) polyethylene terephthalate (PET) cups (“red party cups”). The diameter of the cup opening was 95 mm and the height 121 mm. Cups were covered with a 127 x 178 mm galvanised steel, flashing shingle to act as a rain cover. During the sampling period each month, trap cups were placed in vertically buried, 150 mm long PVC pipe tubes established as fixed sampling points for the five-year duration of the study. When actively sampling, approximately 50 ml of propylene glycol was added as a killing agent and preservative. To deter small mammals, the entire trap assembly was covered with a 40 x 40 cm sheet of steel wire hex netting (“chicken wire”), held in place with steel spikes.

Of the 60 total traps, 40 were placed in areas of partial tree cover dominated by Gray Pine (*Pinussabiniana*), Interior Live Oak (*Quercuswislizeni*), Blue Oak (*Quercusdouglasii*), and Pacific Poison Oak (*Toxicodendrondiversilobum*) and 20 were placed in grassland dominated by non-natives that included wild oats (*Avena* spp.), barleys (*Hordeum* spp.), vetches (*Vicia* spp.), and Heerman’s Tarplant (*Holocarphaheermanii*) with widely scattered oak and pine trees. Over the five years (July 2014 to July 2019), trap cups were placed in the tubes each month for three nights at or near the period of the new moon. The new moon schedule of sampling was chosen to minimise any differences in night-time luminosity between monthly samples and between shaded and open sites. Given this lunar sampling cycle, 2016 has 13 rather than 12 samples as in other years.

The pterostichine specimen records published here cover the full five-year period (see Data Resources below). The initial sampling period (July 2014 to June 2016) was during a significant multi-year drought. In this initial sampling period, only five specimens of *P.vicinus* and nine specimens of *P.californicus* were collected and only one or two specimens were taken in any given sampling period. As those sample sizes are too small to make any meaningful comparisons, we only use beetles for the study of mating marks that were collected from July 2016 onwards. Although California declared an official end to the drought on 7 April 2017 (CBS News. Associated Press 2017), our period of consideration includes the early drought recovery period, which received substantial rains (cumulative 1044 mm (41.01 inches) for October 2016 to March 2017) and then during years that follow when beetle activity was significantly higher.

To connect beetle activity with environmental conditions, we calculated daily sunlight duration in minutes using the NOAA daily solar calculator (NOAA 2021) and specified our study location coordinates. For the rainfall record, we downloaded precipitation data from the rain tip gauge located at the Marsh Creek Fire Station (MRH), 1.3 km west-southwest of the sampling site (N37.89951, W121.86054) from the California Data Exchange Center (cdec.water.ca.gov).

### Terms

Throughout, we use the terms "marked" and "unmarked" or with or without "mating marks" (or similar phrases) to refer to individual beetles that we have inspected and found to have or lack the specific abrasions of the ventrite or elytra as described herein. Based on the argumentation presented, we are assuming a working hypothesis that, when marks are present, the individual beetle has copulated at some point before being collected. The marks alone do not indicate insemination or egg fertilisation status. Our working hypothesis is also predicated on the assumption that unmarked beetles are virgin, i.e. mating always leaves a mark. Although this assumption has not yet been corroborated by soft-tissue studies, we believe there is a high probability this is the case. Possible avenues of enquiry related to this are briefly discussed in the Conclusions section below.

### Museum and laboratory methods

Samples were pinned, labelled and deposited in the Essig Museum of Entomology, UC Berkeley (EMEC). Identifications of beetles were done by KWW, based on specimens compared to holotype specimens, published keys and descriptions (Casey 1913, Will 2016). All images were taken with a modified Microptics XLT digital imaging system using a Canon EOS D7 camera. Image stacks were then aligned and assembled with Helicon Focus version 5.3.

To determine the status of mating marks for each species, specimens were examined under a daylight-filtered fibre-optic light using a Leica MZ12s stereomicroscope and scored as M+ (male marked, e.g. Fig. 1b, d, Fig. 2) F+ (female marked, e.g. Fig. 1a, c), M- (male unmarked) and F- (female unmarked). Male *P.vicinus* are more lightly marked than *P.californicus* and so greater care to inspect those specimens, by shifting them under the light and using higher magnification, was needed to ensure accurate scoring. Dirty or greasy specimens that had the elytral or last ventrite surfaces obscured were gently cleaned with water, soap water or 70% ethanol (EtOH) using a small camel-hair paintbrush. A few, severely dirty and heavily greased specimens were cleaned by soaking them in hot soapy water for 1 hr before being rinsed with clean water.

To estimate the body size range of *Pterostichus* Bonelli species, we used a measurement of standard body length (sbl) in millimetres, calculated as the sum of head length (length from base of the labrum to estimated base of head) + pronotal length (length along the mid-line of the pronotum) + elytral length (length of left elytron from the base of the scutellum to the elytral apex). All individuals were measured using a microscope ocular reticle. The subsample of *P.vicinus* and *P.californicus* that were measured includes the apparent largest and smallest individuals from each set of marked males, unmarked males, marked females and unmarked females, to ensure the full-size range was reported. To calculate the size statistics, four random specimens in each category were selected for a total of 24 specimens per species. For *P.angustus* and *P.protensiformis*, the largest and smallest male and female were measured and five males and five females were randomly selected to calculate the size statistics.

To determine if these mating marks occurred outside of the four species of *Pterostichus* from the Perkins Canyon pitfall trap study, we made a careful examination of the EMEC specimens of the two eastern *Hypherpes* species, *P.adoxus* (Say, 1823) (N = 45) and *P.tristis* (Dejean, 1828) (N = 50), that were treated in Bousquet’s (Bousquet 1986) life cycle study. We also made a cursory examination of some exemplars of North American Pterostichini species represented in the EMEC that had more than 25 specimens and confident identification. The cursory survey was not intended to be exhaustive and was focused on species of *Hypherpes* and a few common species of other *Pterostichus* subgenera. Unit trays of pinned specimens were visually scanned under the microscope, looking for marks on the elytra of female specimens and males randomly selected to view the ventral surface. Specimens were not cleaned or otherwise manipulated.

### Data Analysis

**Figure 3.**
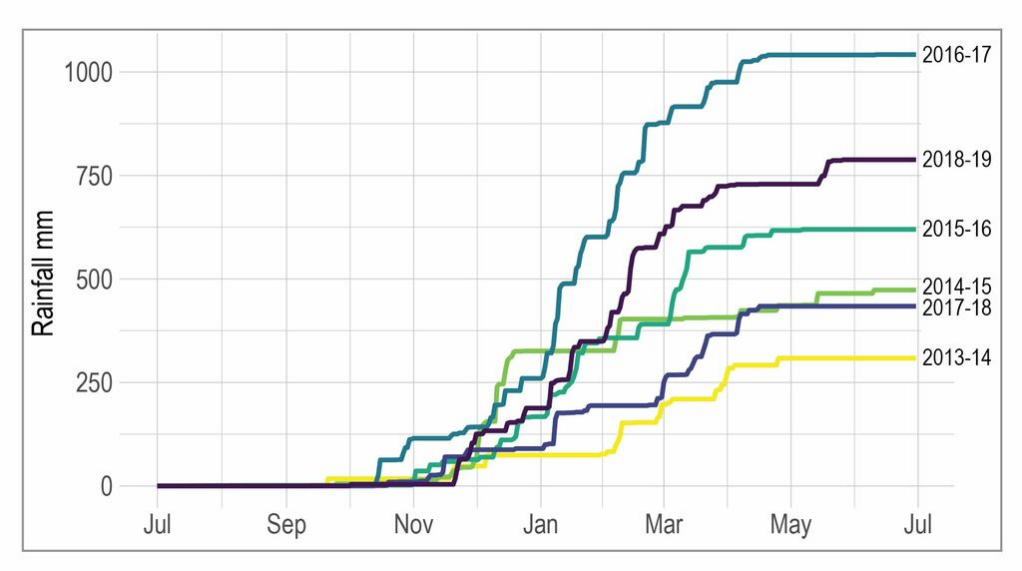
Rainfall summary. Cumulative rainfall for sampling years 2013 to 2019. July 2019 was omitted as no additional rain was recorded during that month

**Figure 4.**
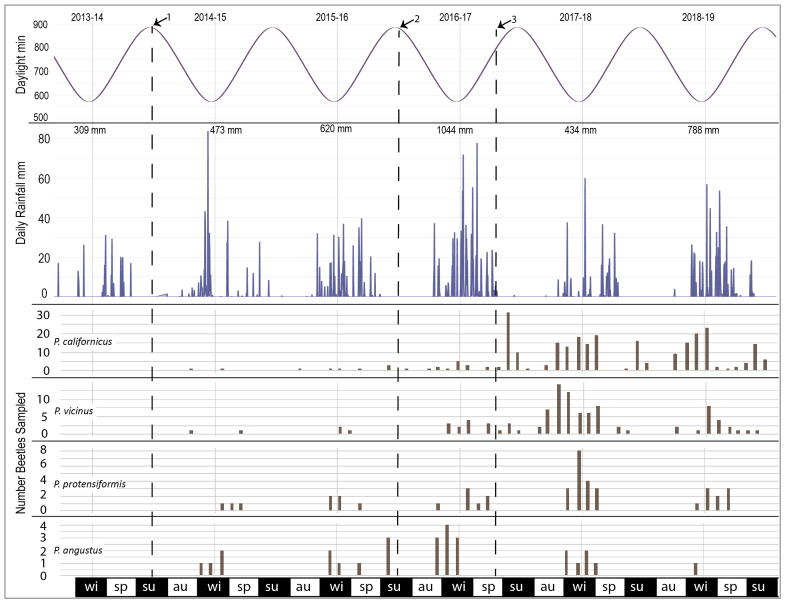
Five-year overview of seasons, photoperiod, rainfall and samples of the four *Pterostichus* species. Dashed lines indicate the following dates: 1 - initiation of sampling for the project; 2 - beginning of the period for which samples are considered for the mating marks study (i.e. the beginning of the drought recovery period); and 3 - the official, State of California end of the drought. Seasons are noted as wi= winter, sp= spring, su= summer, au= autumn.

To characterise adult activity during and after the drought, we tallied the total number of all adult individuals by species, including noting tenerals, for each new moon sampling event. We plotted these counts to create a sequential temporal profile for each species and compared these temporal profiles to the plots of photoperiod, daily and cumulative rainfall (Figs 3, 4). These graphs were used to interpret the influence of photoperiod on adult activity, how the onset of rainfall and total year’s rainfall during the current and prior year was reflected in each year’s cohort. To characterise the seasonal pattern and derive a general pattern of phenology, we plotted all years on the same graph as overlapping years from July 1 - June 30 (Fig. 4) to identify onset, peak and decline of activity. We used these patterns along with the presence of teneral adults to derive when it is likely that larval maturation and periods of adult quiescence occur to characterise the breeding patterns over the course of the seasons as they compare to canonical spring/autumn breeding patterns in the published literature.

To look for a habitat preferences, the per-trap catch of *Hypherpes* species was calculated by dividing the total catch for each species in the two habitat types, oak/pine and grassland, by the number of traps deployed in each of those areas. As P. (Leptoferonia) angustus is known from previous, extensive hand-collection to be mostly restricted to drainages adjacent to the sampling sites, its occurrence is considered extralimital and the records for this species are not included in the habitat preference calculations.

To further understand the reproductive periods of these species, we calculated sex ratios using the abundance of males and females of each species. We estimated the temporal pattern of the mated and unmated individuals each year using the scores derived from the mating marks. Although these marks do not reveal if fertilised eggs were deposited, we can confidently identify which individuals in the population that have previously copulated or not prior to the time of the sampling events. As the sampling period included the end of a multi-year drought with very low abundance of individuals, the focus for the interpretation of the mating marks on the life history is on the material collected after July 2016 just prior to the 2016–2017 rainy season, which we consider the drought recovery period.

## Data resources

Specimen data for all four *Pterostichus* species are publicly available in UC, Berkeley, Essig Museum's EssigDB and in DataDryad at https://doi.org/10.6078/D1B40R.

## Results

### Habitat conditions

Total precipitation from 1 July through to 30 June of the following year varied with drought years receiving under 500 mm of precipitation and non-drought years receiving up to 1000 mm (Fig. 3). The only exception for a non-drought year was 2017-2018, which only received 434 mm, which was more similar to a drought year in terms of total precipitation. With the exception of 2016-2017, where 115 mm of rainfall accumulated by 1 Nov 2016, all other years only began to receive low levels at this point, accumulating almost half of the year’s total rainfall by the end of January. Nearly all years received all of their rainfall by the end of April or May, often punctuated by a major rainfall event of 28-68 mm accumulated over several days (Fig. 4).

### Sampling results

During the sampling period from July 2014 to June 2019, a total of 437 specimens of the four *Pterostichus* species were collected (Fig. 4) with *P.californicus* (N = 267) and *P.vicinus* (N = 100) as the dominant species (Table 1) and *P.protensiformis* (N = 42) and *P.angustus* (N = 28) substantially less common (Fig. 4). Of the total 437 specimens, only about 8% of the individuals were collected in the first 24 months that preceded the end of the multi-year drought period (July 2014 – June 2016). The drought period samples include only 33 specimens: *P.vicinus* (N = 5), *P.californicus* (N = 9), *P.protensiformis* (N = 8) and *P.angustus* (N = 11). Pitfall traps captured *P.californicus* in equal numbers in oak/pine and grassland habitats and yielded at least twice as many individuals per trap compared to other species (Table 2). Oak/pine pitfall traps recovered more *P.vicinus* and *P.protensiformis* than grassland traps.

**Figure 5.**
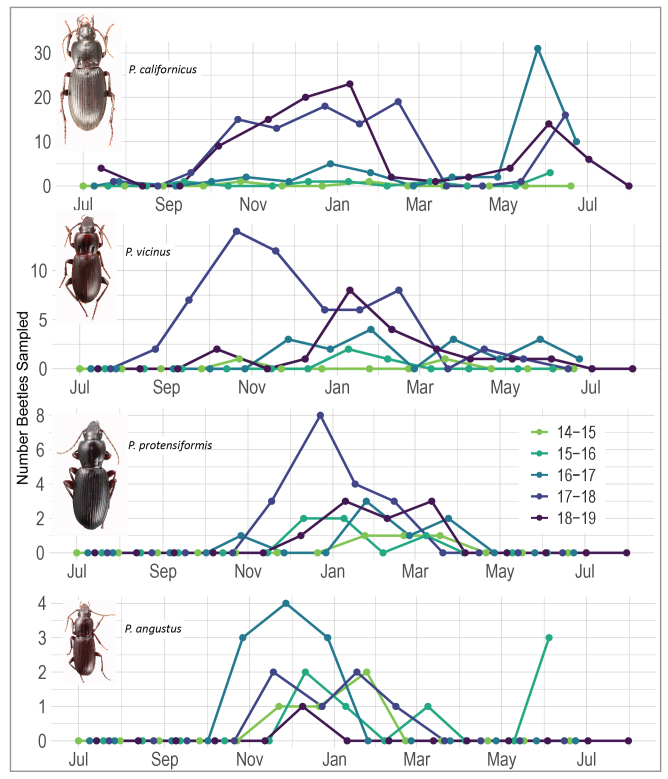
Samples by *Pterostichus* species over the entire sampling period shown with overlapping years to emphasise repeated periods of activity.

From the beginning of the 10-month drought recovery period (July 2016 – April 2017) until the end of the study in 2019, the maximum single sample take of the dominant species was 30 individuals in May 2017 for *P.californicus* and 14 individuals in October 2017 for *P.vicinus* (Fig. 4). Records for both species during the drought period indicated low activity or population size that increased during the first year after the 2016-2017 wet season that brought an end to the multiple-year drought. For *P.californicus*, from July 2017 onwards, adult activity peaks twice with a long period of adult activity from mid-September through March (2018) or February (2019), followed by a short adult activity period concentrated in May–June (Fig. 5). For *P.vicinus*, total abundance is ~ 1/3 lower than *P.californicus.* Activity in years following the drought occurred earliest in 2017–2018 beginning in August 2017 and peaking by late October. All other post-drought years for *P.vicinus* had an activity that began 1–2 months later. Teneral specimens were uncommon, but identified for *P.californicus* and *P.vicinus* in all peak activity periods throughout the year except for the rainy season period in late November to January (Table 3).

The other two species had a lower abundance with a maximum of eight individuals of *P.protensiformis* in December 2017 and four individuals of *P.angustus* in November 2016. For *P.protensiformis*, numbers of individuals were generally low, but their presence was detected in pitfall traps between late October and April, usually beginning in the month following the first substantial rains of the season, even in drought years. For *P.angustus*, adults were similarly detected between October and April, albeit at even lower numbers, even during the drought in late 2014 and 2016, but were not found in pitfall traps after January in the two very wet years with high January–March rainfall totals (2016–2017, 2018–2019). Tenerals of *P.protensiformis* and *P.angustus* did not appear in pitfall traps.

### Body lengths

Body lengths of the larger species, *P.californicus* (mean sbl = 15.6 mm, range 13.5–17.7 mm), *P.protensiformis* (mean sbl = 15.1 mm, range 13.7–17.8 mm) and *P.vicinus* (mean sbl = 13.6 mm, range 12.3–14.5 mm) broadly overlap; however, the mean length of *P.vicinus* is 1.5–2.0 mm smaller than the other two species. Relative to these three *Hypherpes* species, *P.angustus* (mean sbl = 9.3 mm, range 8.3–10.1 mm) is typically a full third smaller.

### Sex ratios

Sex ratios for *P.californicus* and *P.protensiformis* were close to 1:1 for males:females. In terms of the monthly sex ratio, in *P.californicus*, the sex ratio skewed towards females in months with large samples of beetles (May 2017, February 2018 and June 2018). For *P.vicinus*, females were almost twice as common in the samples as males across the entire study period (Table 1). This skew for *P.vicinus* is mostly a result of a strong female bias from July to November of 2017. For *P.angustus*, the species with the fewest overall observations in the dataset, the sex ratio was somewhat skewed towards males.

### Occurrence of mating marks

**Figure 6.**
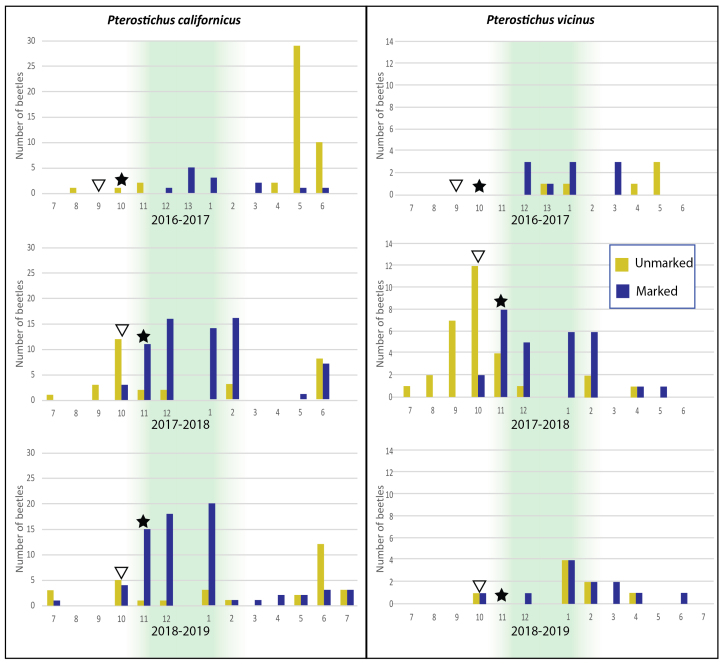
Summary of Perkins Canyon pitfall trap catch counts for *P.californicus* and *P.vicinus* with presence or absence of mating marks pooled for males and females indicated by bar colour for each sampling period. Green shaded periods are the typical core precipitation periods in California. Triangles indicate the month with the first measurable rainfall. Stars mark the first month each year with > 63 mm (~ 2.5 inches) of cumulative rainfall. As samples were taken at or near the new moon, 2016 has 13 sampling periods and 2017–2018 having only 12.

Mating marks were found in some individuals of males and females of both *P.californicus* and *P.vicinus*. For these species, the ratio of F+ to F- was about equal overall, while M+ were almost twice as frequent as M- (Table 1). For *P.californicus*, the largest sample and percentage of unmarked individuals occurred after the first winter following the wet winter that ended the drought in May of 2017 (Fig. 6). Unmarked and presumably unmated individuals generally appear in May or June of each year, are absent in August, then reappear and increase in abundance again in October. In October, based on the appearance of marks, mating begins and continues throughout the end of the year with upwards of 85% marked by the November sample and 90% by December in 2018 and 2019. For *P.vicinus*, unmarked individuals increase in abundance from May through October of 2017, after which 70% show mating marks as of November and 80% by December (Fig. 6). Beyond March 2018, the sample size is very low with both marked and unmarked individuals appearing in most months. In both species, there is a notable bias towards unmarked individuals in samples taken in the April through October samples in 2017 following the end of the drought. The other two species in this study, *P.protensiformis* and *P.angustus*, lack mating marks, making their mating status unknown.

The position of the mating marks on specimens of *P.californicus* and *P.vicinus*, when present, is invariably found in the apical third of the elytra on intervals 1–3 in females and in the apical half of the last ventrite of males.

Females of both species and males of *P.californicus* were always either unambiguously marked or unmarked. In *P.californicus* females, the mark is an abrasion that varies from a patch of longitudinal scratches at the level of the elytral plica and restricted to the first interval, to a deeply excavated concavity on the first interval that is progressively shallower laterally, extended on to the second interval (Fig. 1a). In some cases, the abraded region can reach the third interval. The marked area is roughly centred on the elytral suture. *Pterostichusvicinus* females are notably different in the position of the mark, which is very near the apices of the elytra, beyond the level of the plica (Fig. 1c). Like *P.californicus*, the depth ranges from a patch of scratches to deep abrasions revealing the paler exocuticle below. However, it is typically more heavily abraded on the left elytron rather than consistently medial.

Males of both species are much less deeply marked than females, marks are represented by a distinctly scraped region that usually completely obscures the reticular microsculpture of the unmarked cuticle, but never eroded enough to reveal the paler exocuticle. In all marked males, the region of the last ventrite is distinctly scratched and the position is very consistent within species (Figs 1b, d, 2), typically slightly left of the mid-line. *Pterostichuscalifornicus* males have a mating mark patch that is roughly round (Fig. 2a) and is about a third of the median length of the ventrite from the apical margin. The mark in *P.vicinus* males is typically less distinct than *P.californicus* and somewhat elongate or roughly shaped like a "7" (Fig. 2b) and often starts quite close to the apical margin.

For other species examined, the variation of the position and form have not been recorded in detail. However, they all occur in the apical third of the elytra on intervals 1–3 in females and in the apical half of the last ventrite of males. Twelve species of *Hypherpes* in addition to *P.californicus* and *P.vicinus*, were found to bear mating marks in males and females: *P.algidus* LeConte, 1853, *P.amethystinus* Mannerheim, 1843, *P.crenicollis* LeConte, 1873, *P.gliscans* Casey, 1913, *P.illustris* LeConte, 1851, *P.inermis* Fall, 1901, *P.jacobinus* Casey, 1913, *P.ordinarius* Casey, 1913, *P.protractus* LeConte, 1860, *P.serripes* (LeConte, 1875), *P.tarsalis* LeConte, 1873 and *P.tuberculofemoratus* Hatch, 1936.

Eleven species examined had no individuals with mating marks. Neither of the eastern *Hypherpes* species, *P.adoxus* and *P.tristis*, had mating marks, nor were marks found on Perkins Canyon specimens of *P.protensiformis*. The following species from subgenera other than *Hypherpes* also lacked mating marks: P. (Bothriopterus) mutus (Say, 1823), P. (Leptoferonia) angustus, P. (Leptoferonia) inanis Horn, 1891, P. (Leptoferonia) sphodrinus LeConte, 1863, P. (Petrophilus) melanarius (Illiger, 1798), P. (Petrophilus) stygicus (Say 1823), Cyclotrachelus (Evarthrus) sigillatus (Say, 1823) and *Poecilusdiplophryus* Chaudoir, 1876.

## Discussion

### *Pterostichus* of Perkins Canyon

The sampling area, Perkins Canyon, is a mix of grassland and light, oak and gray pine woodland. Four species of *Pterostichus* are known from the area and all appear in the trap samples.

Two species, Pterostichus (Leptoferonia) angustus and Pterostichus (Hypherpes) protensiformis, neither with apparent mating marks, are only represented by a relatively small number of specimens from the five-year study. Only 28 specimens of the small-sized (sbl 8.3–10.1 mm) *P.angustus* were collected and nearly all of those were during the October to March periods (Fig. 4). The activity of adults of this species tracks closely the cooler, short-day, rainy season. *Pterostichusangustus* is commonly hand-collected across its range during the rainy season and well into spring in relatively moist ravines with a fairly closed tree canopy and abundant leaf litter. This type of habitat is only at the margins of our sampling sites, which may account for the small number of specimens taken in traps. A total of 42 specimens that have been provisionally identified as *P.protensiformis* were sampled in winter and early spring periods and, like *P.angustus*, were active during the cooler, short-day, rainy season. It must be noted that identification remains provisional for these beetles as species of *Hypherpes* have not been revised and specimens that fit Casey’s description of this species are polymorphic for size (sbl 13.7–17.8 mm, but with distinctly large, broad specimens and small, narrow specimens) and pronotal form. *Pterostichusprotensiformis* is sympatric with *P.californicus* and *P.vicinus* and, based on hand collecting as well as the pitfall trap samples, is consistently less abundant than either of those species. Like *P.vicinus* and *P.californicus*, as discussed below, the abundance of *P.protensiformis* seems to correspond to the previous rainy season’s rainfall totals (Fig. 4).

The four sympatric and synchronically active *Pterostichus* species undoubtedly interact, but are at least in part, apparently separated by size, secondary sexual characters and habitat preferences. *Pterostichusangustus* is unlikely to have significant interactions with the three *Hypherpes* species as it is both much smaller and has a distinctly different habitat preference as described above. *Pterostichusprotensiformis* and *P.vicinus* were much more common in traps placed in the oak and pine habitat than they were in the grassland traps, while *P.californicus* was sampled equally across both the tree-covered and grassland habitats (Table 2). Amongst these three *Hypherpes* species, *P.vicinus* is typically smaller, males have a prominent ventral denticle of the hind femur not present in the other species and it shows a preference for tree-covered habitat. Presumably, this combination of attributes helps to keep these similar and closely related species ecologically separated.

Of the four Perkins Canyon species, only *P.vicinus* samples show a sex ratio bias, which is female dominant overall (Table 1). However, this is almost entirely due to a large number of unmarked females appearing in traps during the initial, post-drought period (Figs 4, 6) and this is not obviously the case over the remaining sampling period.

### Seasonality and breeding patterns in *P.vicinus* and *P.californicus*

All Perkins Canyon *Pterostichus* species are far less active during periods of extended drought and the number of individuals taken during the drought period early in this study (2014–2016) is too small to detect any particular pattern (Figs 4, 5) beyond that; what little activity detected was coincidental with rainy seasons. In the 2016–2017 core rain period, when the drought broke, there was a noticeable increase in activity, but far fewer beetles were taken than in the same season in the subsequent non-drought years (Fig. 4). In the spring and summer following the drought, there is an apparent rebound in adult activity. Overall the sampled *Pterostichus* species are taken in greater numbers in the autumn and through winter, during the core rain period, but activity, as represented by increasing trap take, usually begins prior to the first month of significant rainfall amounting to an accumulation of > 2.5 inches (63.5 mm) over a single sampling month, at or just prior to the first measurable rainfall (Fig. 6).

There are distinct peaks of adult activity in *P.californicus* in early autumn through winter rainy season and then again in late spring to early summer (Figs 4, 5, 6). Late summer-autumn adult activity begins prior to the onset of significant rains (see stars in Fig. 6) and this and the spring activity are probably initiated by the photoperiod (Fig. 4). Based on the appearance of mating marks, mated and unmated individuals are present all year, but shift from a high ratio of unmated before November of each year to nearly all individuals showing evidence of having mated throughout April of each year until new unmated individuals reappear in June. The sex ratio stays close to 1:1 (Table 1).

The seasonality of *P.vicinus*, like *P.californicus*, peaks in the rainy season in autumn through winter. There is some activity in late spring to early summer (Figs 4, 5, 6), but it does not appear as prominently. Similarly, autumn adult activity begins prior to the onset of significant rains. The presence and absence of mating marks show that mated and unmated individuals are present much of the year. The sex ratio in samples is notably or somewhat female-biased over the entire sample (Table 1). However, the magnitude of the bias toward females is largely due to the relatively large number taken after the drought broke in 2017 and this pattern largely dissolves in the final year of the study (2018-2019) as the number of beetles sampled returns to low levels, perhaps in response to the low rainfall in the winter of 2017-2018. This species may require a more moist habitat as it was found twice as often in the pitfall traps in the oak/pine habitat.

Mating-marked individuals, presumably the previous year’s females, were present and active in *P.californicus* and *P.vicinus* in late winter and early spring 2017. Marked and presumably previously mated individuals were rare for *P.californicus* and absent from the samples of *P.vicinus* from April until October of 2017. The large percentage of unmarked individuals of both species during this period suggests that there was a response to the accumulated precipitation ending the drought by the emergence of a mostly unmated cohort of adults.

Discerning these patterns was enhanced by the 5 years of monitoring which included both drought and recovery years. The activity abundance of *P.vicinus* and *P.protensiformis* and, to a lesser degree, *P.californicus*, appears to correlate less with the same-year rainfall than with the quantity of previous winter's rains. For example, we see an increased trap catch in spring 2017 and during the 2017–2018 rainy season following the relatively robust rainfall total in 2016–2017 (1044 mm), even though the 2017–2018 rainfall totals are at drought-level (434 mm). Subsequent to the poor 2017–2018 rainy season, *P.vicinus* and *P.protensiformis* numbers dropped noticeably and *P.californicus* numbers modestly declined. The latency of activity apparent in the samples might be all or in part due to: (1) the previous year's adults that aestivated the prior summer or (2) eggs laid and larvae that developed in the previous autumn-winter, which then pupated in late winter-early spring, speeding up development to emerge and synchronise the adult's activity period (dynamic phenotypic polyvariance sensu Makarov (1994)). Either of these scenarios would be consistent with the large number of unmated individuals in spring and early autumn 2017. The capture of this phenomenon further highlights the role of long-term datasets (> 5 years) for understanding the role of inter-annual variation in environmental conditions on life cycle timing (Jackson and Fureder 2006).

The pattern we found for these species does not easily fit the canonical spring-breeder/autumn-breeder pattern that is often used to describe wet-temperate regions. The patterns of both *P.californicus* and *P.vicinus* are consistent with a life cycle that is typically annual, univoltine (but possibly facultatively biennial semi-voltine in dry years), rainy season (autumn-winter) iteroparous, with adult aestivation and facultative larval hibernation. However, unmarked and presumably unmated individuals and teneral adults were captured during peak activity periods regardless of season (Table 3), suggesting that either the beetles diapause as pupae or teneral adults that complete development and become active at various points during the year and/or there are at multiple periods of breeding and oviposition each year, in at least some portion of the population. Mixed populations of overlapping generations is a strategy that Kaufmann (1971) demonstrated in Alaskan *Pterostichus* (*Cryobius) brevicornis* (Kirby) and that is known or thought to occur in many *Pterostichus* species (Bousquet 1999) and more widely in Pterostichina, for example, in species of *Poecilus* Bonelli (Storre et al. 1996).

Perkins Canyon is located in the California Mediterranean-type climate region, where the weather regime is characterised by cool, wet winters and hot, dry summers, which differs from the generally wet, strongly seasonal, temperate habitat that most published accounts of pterostichine life history are based. Across all of these environmental cues, autumn activity may be initiated by the photoperiod, while oviposition and larval development timing are likely to be closely synchronised in response to the Mediterranean-type climate wet season given that egg development in carabids is thought to require moisture (Paarmann 1986) and larvae are sensitive to desiccation (Paarmann 1973). In addition, it is possible that pupation and adult emergence of the new cohort may not occur until after the last intensive rains of the season in late April or May of each year when the surface soils in the landscape begin to dry and vegetation dies back.

### Mating marks

In the process of identifying specimens, we initially noticed that *P.californicus* had characteristic wear-marks on the elytra and that these marks were on many specimens in the same position. The marks vary from noticeable scuffs or a series of scratches to deep, flat or concave abrasions into the elytral intervals that expose the pale, internal exocuticle. In the past, we had assumed that these were simply marks left from some environmental source, such as abrasive materials in the substrate the beetles live in, predation attempts or specimen handling during preparation. However, it would be expected that these various, potential sources of the marks would leave marks more or less randomly on the cuticle and that such marks would be on both sexes equally, which was not the case in *P.californicus*. Elytral marks are exclusively on females and always in the same position (Fig. 1a).

There are two likely causes for such marks on the female elytra, oviposition or mating. To our knowledge, there is nothing that would suggest *Pterostichus* females have a behaviour during egg-laying that would produce such a mark. On the other hand, *Pterostichus* males, like all carabids, grasp the female from the dorsal side and are orientated in the same direction as the female while copulating. They primarily grasp the female around the mesothorax with their middle legs and hold dorsally on to the female’s pronotum with their expanded protarsi. In this position, the male’s last ventrite is in contact with the female's elytra. The specific elytra abrasions in females then predict a complimentary mark on the males’ last ventrite. Such marks were found (Figs 1b, d, 2) and are only found on males. Although marks on the male ventrite are never as deeply worn as in females, they are consistently a dull, worn region that is clearly evident and uniformly placed. Notably, teneral specimens of both sexes very rarely have any marks (Table 3).

Having confirmed the occurrence of the marks in *P.californicus*, we investigated *P.vicinus*. In *P.vicinus*, we found similar marks as in *P.californicus*; however, the location of the marks in the females was clearly different in the two species (Fig. 1a, c). In *P.californicus*, the mark is on the first two elytral intervals at about the level of the elytral plica and, in *P.vicinus*, those same intervals are marked, but well beyond the level of the plica, quite near the apices of the elytra. The difference in the position of the marking is also consistent with the differences in the male genitalia. The apical lamina (tip) of the median lobe of the aedeagus in *P.californicus* is very elongate, while the apical lamina of *P.vicinus* is very short. The longer the lamina, the further forward on the female the male can be positioned and still reach the female’s genitalia and the further anterior the mark will be produced.

Therefore, these marks must be produced during courtship, mating or after mating, during mate-guarding. Whether there is courtship, a period of mate-choice prior to copulation or a post-copulatory mate-guarding is not known. Although courtship and mate-guarding behaviour have not been confirmed in any *Hypherpes* species, we have frequently seen beetles in the field with the male holding the female in a typical mating position, but without genital copulation. Given the correlation of the mark’s position with the male genitalia form, however, it seems likely that the mark is produced during copulation.

Frequently the elytral marks on the females are very deep and, as such, suggest a significant amount of force and rubbing is needed to produce the mark. Such deep marks could be made by a single male during a long-duration copulatory episode or females may be polyandrous and the mating mark is made more substantial by each mating encounter.

In our limited survey of taxa, mating marks were only found in species of *Hypherpes.* We only report here results from species that were represented by a sufficient number of specimens to feel confident that there is a strong indication of the presence or absence. In addition to species of various *Pterostichus* subgenera, other than *Hypherpes*, that we found lack mating marks, we have casually looked at specimens of many different tribes of carabids and have not found mating marks. Perhaps this phenomenon is restricted to *Hypherpes*, but given the vast number of Carabidae to be examined, we consider the possibility that marks like these occur in other taxa as very high. A novel evolution or perhaps repeated gains and losses within *Hypherpes* would be consistent with our findings that species in the subgenusLeptoferonia, which is part of the *Hypherpes* complex (Will and Gill 2008), lack mating marks, as do the eastern *Hypherpes* species, *P.adoxus* and *P.tristis*. However, the subgenusHypherpes is in need of revision, a more robust phylogeny and a comprehensive survey for mating marks to fully understand the pattern of evolution.

The mating marks are presumed to indicate mating behaviour that involve the contact of the male ventrite and female elytra. In some *Hypherpes* species males and males of various other *Pterostichus* species, the last ventrite is modified, frequently as a raised area or by the presence of one or more large tubercles (Hacker 1968,Bousquet 1999, Lindroth 1966). How these secondary sexual characteristics of the ventrite are related to mating marks and behaviour is unclear. Within our small sample of taxa, we found both of the sister species pair (Will and Gill 2008) *P.serripes* and *P.tarsalis* have mating marks. *Pterostichusserripes* possesses a large, keel-like tubercle on the ventrite and *P.tarsalis* entirely lacks this tubercle, but has a slight medial concavity. The last ventrite of *P.californicus* has only a very slightly raised region and *P.vicinus* is unmodified and both have mating marks. *Pterostichusadoxus* and *P.tristus* vary from an unmodified male ventrite to having a notable, but low, median ridge, but both species were found to be unmarked.

## Conclusions

The mating marks we report here for 14 species of *Hypherpes* represent the only occurrences of this particular phenomenon of external cuticular abrasions revealing apparent mating status known for insects. However, it would not be surprising to find similar kinds of indications of mating or other behaviour on museum specimens in other carabids or even other insects. We encourage efforts to look carefully for such characteristics during studies like monographic revisions, taxonomic descriptions and other uses of museum specimens. As museum collections extend back many decades, we anticipate that more extensive surveys of holdings may reveal stasis and change in the breeding and activity patterns relative to drought and fire, two of the most prominent forces in the Californian Mediterranean-type climate. Ongoing, long-term monitoring projects may also benefit from surveying mating marks as an easily accessible indication of population dynamics.

In the present case, we were able to combine seasonal abundance and mating marks to help provide information for our understanding of the life history of two pterostichine species by using it as a tool to identify when new, unmarked (unmated) adults were recruited into the population and, based on the appearance of the marks, when most individuals had mated at least once. The beetles' life history likely evolved over the last 3 Ma period that established the Mediterranean-type climate in the region (Bartolome and Spiegal 2020). As a result of the high inter-annual variation in precipitation in California, it appears that there are mixed populations of overlapping generations, a hallmark of phenotypic polyvariance.

Many questions remain for further study. For example, what is the distribution of this phenomenon on the phylogeny of *Hypherpes* species and across habitats where the beetles occur? How do the mating marks relate to male aedeagus and paramere structures and male secondary sexual characteristics, such as tubercles on the ventrite or dentate femora? In females, how do the marks correlate with the condition of the ova, corpora lutea and presence of sperm in the spermatheca? In addition, what exactly, is the specific behaviour that produces the markings?

## Figures and Tables

**Figure 1a.**
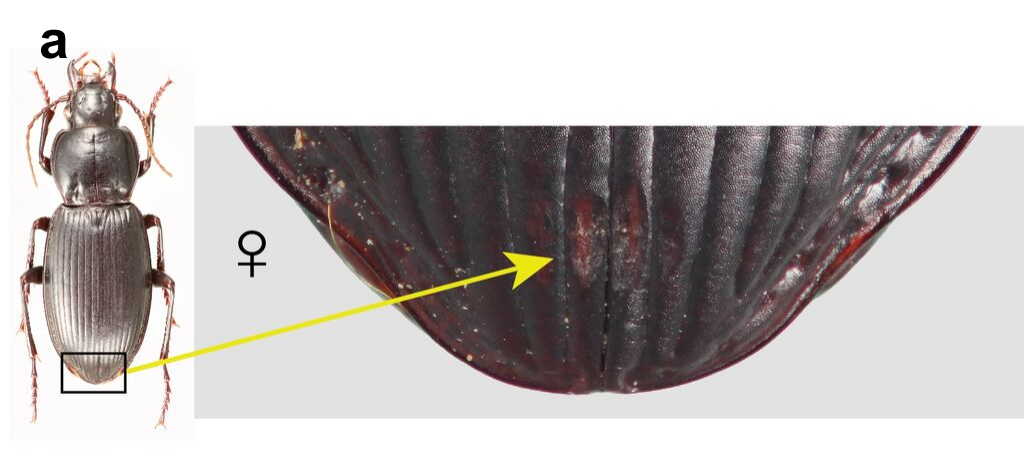
Dorsal habitus of *Pterostichuscalifornicus* female (left) and elytral apices with mating marks (right). Box indicates the regions that the mating marks are located and arrow indicates the marks in close-up.

**Figure 1b.**
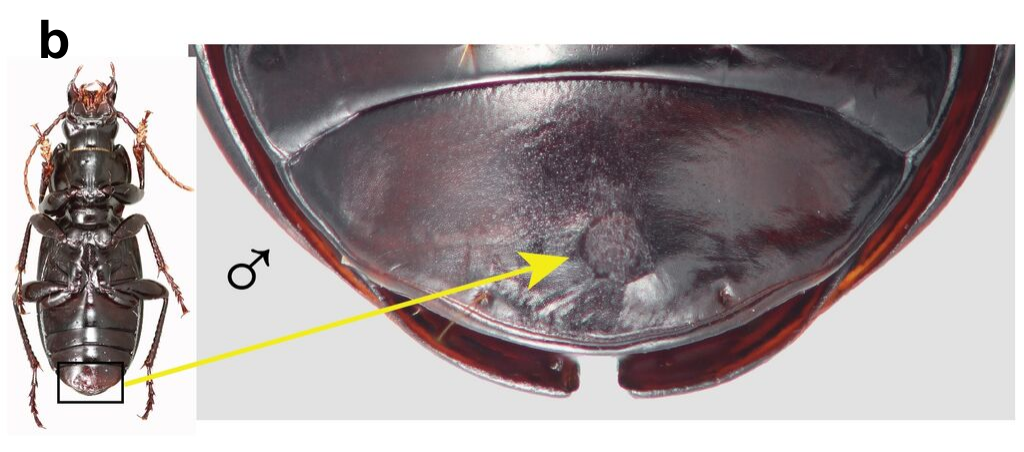
Ventral habitus of *Pterostichuscalifornicus* male (left) and apical ventrite with mating marks (right). Box indicates the regions that the mating marks are located and arrow indicates the marks in close-up.

**Figure 1c.**
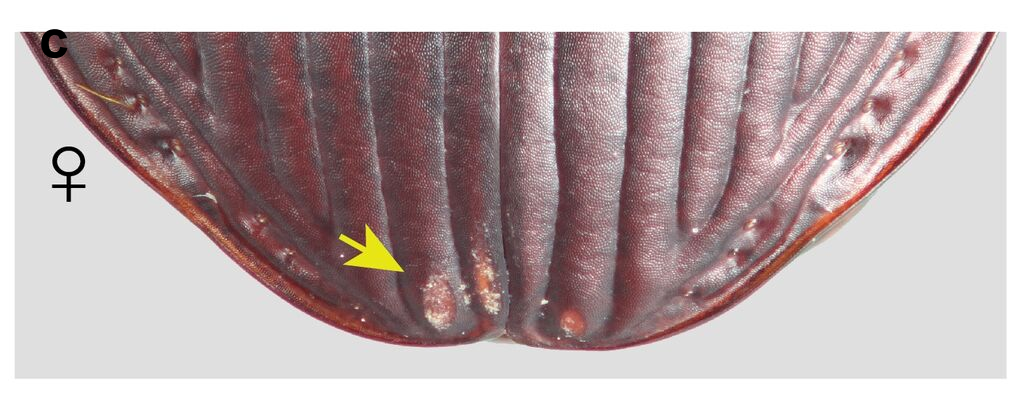
The elytral apices of female *P.vicinus* with mating marks.

**Figure 1d.**
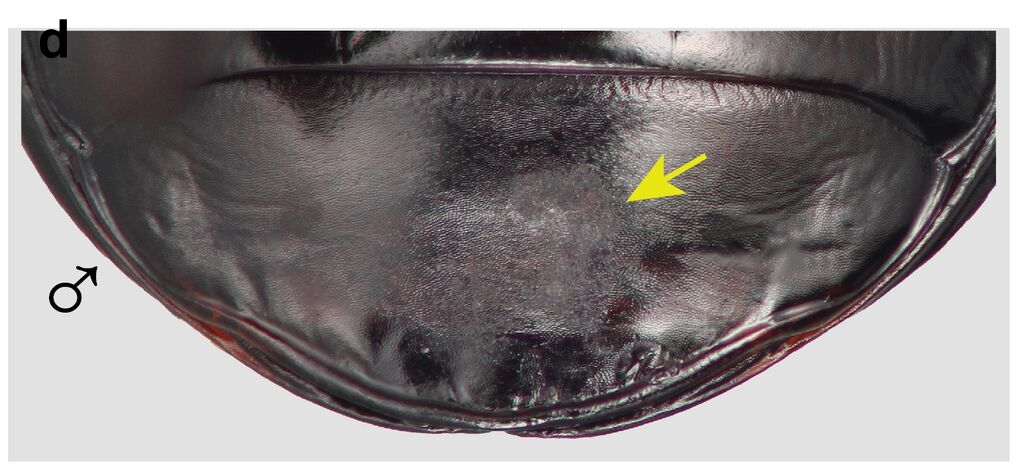
The last ventrite of males with mating marks for *P.californicus* and **D**, *P.vicinus*.

**Figure 2a.**
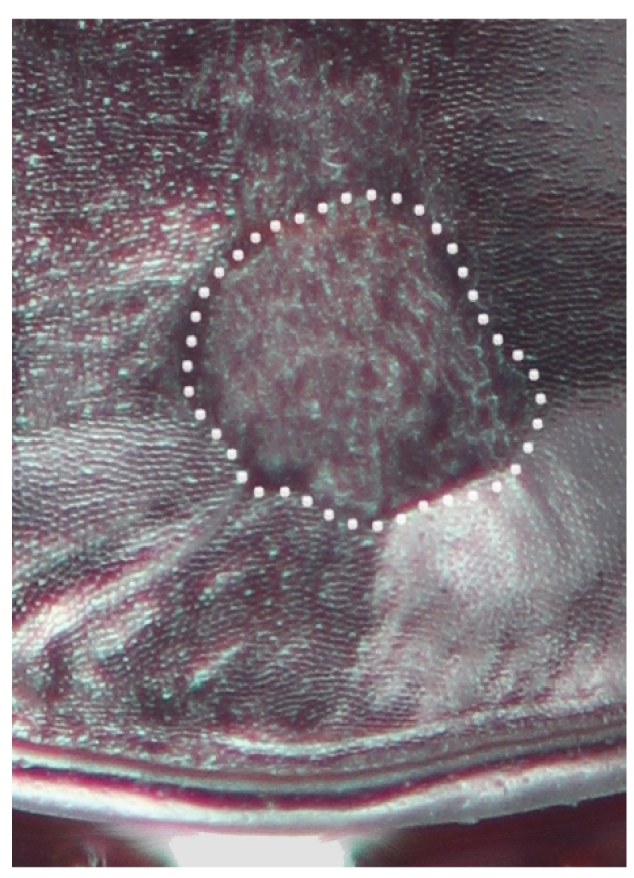
Pterostichus californicus

**Figure 2b.**
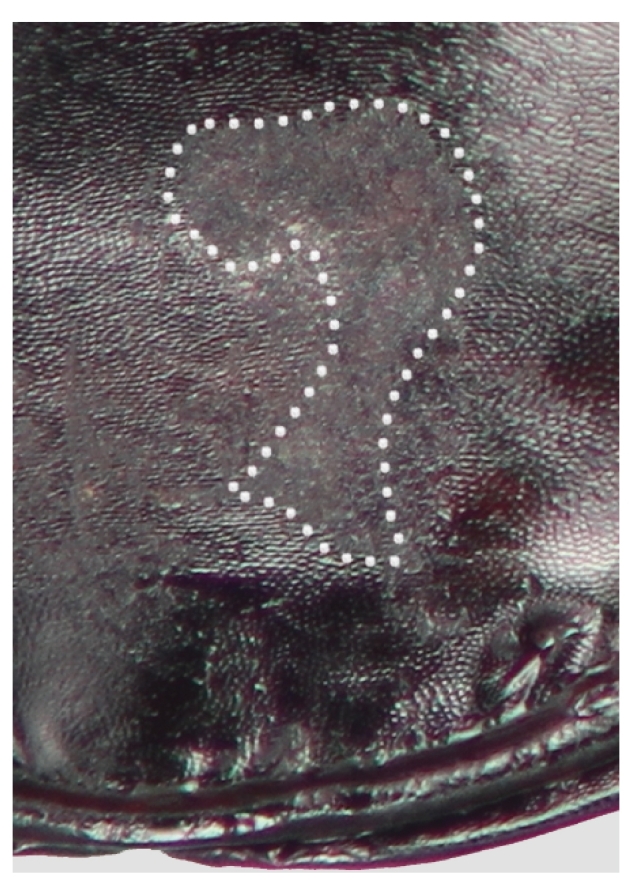
Pterostichus vicinus

**Table 1.** Counts of females (F), males (M) and marked (+) or unmarked (-) individuals from the Perkins Canyon samples of *Pterostichus* species included in this study. Boxes with “na” indicate that the species lacked mating marks on all material examined.

**Species**	**Total**	**Female**	**F**+	**F**-	**Male**	**M**+	**M**-
* P. californicus *	267	131	66	65	136	89	47
* P. vicinus *	100	64	34	30	36	21	15
* P. protensiformis *	42	21	na	na	21	na	na
* P. angustus *	28	11	na	na	17	na	na

**Table 2.** Per-trap catch of *Hypherpes* species by habitat at the trap sites. Forty traps were placed in oak/pine and 20 in grassland.

**Species**	**Oak/pine**	**Grassland**
* P. californicus *	4.4	4.6
* P. vicinus *	2.1	0.9
* P. protensiformis *	1.45	0.45

**Table 3.** The total number of male and female teneral specimens taken in traps summed by month for the entire sampling period.

**Species**	**Total**	**Jan**	**Feb**	**Mar**	**Apr**	**May**	**Jun**	**Jul**	**Aug**	**Sep**	**Oct**	**Nov**	**Dec**
* P. californicus *	8	0	1M+	0	0	1M-,1F-	1M-	1F-	0	0	2M-,1F-	0	0
* P. vicinus *	10	0	1F-	0	3F-	1M-,1F-	0	0	0	2M-,1F-	1F-	0	0
